# Supplementary material for: Content-rich biological network constructed by mining PubMed abstracts
Source: BMC Bioinformatics. 2004 Oct 8;5:147. doi: 10.1186/1471-2105-5-147 (PMC528731; doi:10.1186/1471-2105-5-147)
Supplement: Additional File 5 — The original Chilibot query results of the term "long-term potentiation (LTP)" and 22 other terms, limiting the latest references analyzed to the years 1990, 1995, 2000, and 2004. [file 1471-2105-5-147-S5.bz2 › chilibotAdditionalFile5/ltp2000/html/TRKA_TAU.html]

 


 **TRKA** and **TAU** 
  
Found 1 abstracts in PubMed,  **1 abstracts were retrieved and analyzed**.  


---

 Search Google  |
 PDF files only 
|  EDU domain only 

---

- J Neurosci Res, 2000   Bone marrow stromal cells exhibit multiple traits of a stem cell population.
  They can be greatly expanded in vitro and induced to differentiate into multiple mesenchymal cell types.
  However, differentiation to non mesenchymal fates has not been demonstrated.
  Here, adult rat stromal cells were expanded as undifferentiated cells in culture for more than 20 passages, indicating their proliferative capacity.
  A simple treatment protocol induced the stromal cells to exhibit a neuronal phenotype, expressing neuron specific enolase, NeuN, neurofilament M, and **tau**.
  With an optimal differentiation protocol, almost 80% of the cells expressed NSE and NF M.
  The refractile cell bodies extended long processes terminating in typical growth cones and filopodia.
  The differentiating cells expressed nestin, characteristic of neuronal precursor stem cells, at 5 hr, but the trait was undetectable at 6 days.
  In contrast, expression of **trkA**, the nerve growth factor receptor, persisted from 5 hr through 6 days.
  Clonal cell lines, established from single cells, proliferated, yielding both undifferentiated and neuronal cells.
  Human marrow stromal cells subjected to this protocol also differentiated into neurons.
  Consequently, adult marrow stromal cells can be induced to overcome their mesenchymal commitment and may constitute an abundant and accessible cellular reservoir for the treatment of a variety of neurologic diseases.
